# Supplementary material for: Downregulation of SOX9 expression in developing entheses adjacent to intramembranous bone
Source: PLoS One. 2024 May 10;19(5):e0301080. doi: 10.1371/journal.pone.0301080 (PMC11086909; doi:10.1371/journal.pone.0301080)
Supplement: S3 Table — (DOCX) [file pone.0301080.s003.docx]

**Table 3.** Primer assay ID and amplicon length of RT-PCR.

| **Primer** | | **Assay ID** | **Amplicon Length** |
| --- | --- | --- | --- |
| Sox9 | transcription factor for differentiated chondrocyte and tendon cells | Mm00448840_m1 | 101 |
| Runx2 | transcription factor for differentiated osteoblast | Mm00501584_m1 | 91 |
| GAPDH | housekeeping genes expressed in most cells are used as an internal control | Mm99999915_g1 | 107 |
